# Immune Thrombocytopenia Resolved by Eltrombopag in a Carrier of Glucose-6-Phosphate Dehydrogenase Deficiency

**DOI:** 10.4274/tjh.2015.0181

**Published:** 2016-02-17

**Authors:** Laura Scaramucci, Pasquale Niscola, Massimiliano Palombi, Andrea Tendas, Marco Giovannini, Paolo de Fabritiis

**Affiliations:** 1 Sant’Eugenio Hospital, Clinic of Hematology, Rome, Italy

**Keywords:** Idiopathic thrombocytopenic purpura, Glucose-6-phosphate dehydrogenase deficiency, Thrombopoietin mimetic peptide, TMP mimetic peptide

## TO THE EDITOR

Eltrombopag, a thrombopoietin mimetic peptide, may provide excellent clinical efficacy in steroid-refractory patients with immune thrombocytopenic purpura (ITP) [1,2]. Eltrombopag is generally well tolerated. However, its use in the particular setting of glucose-6-phosphate dehydrogenase (G6PD) and history of acute hemolytic anemia (AHA) has not been reported so far. A 51-year-old female was diagnosed as having ITP in September 2014. She was not taking any medication and her past history was negative, apart from having been diagnosed a carrier (heterozygous) of G6PD deficiency (Mediterranean variant) after a familial screening by molecular and biochemical methods. She presented with only slightly reduced (about 50%) enzyme level, belonging to World Health Organization-defined class 3 [3,4]. In the following years, the patient experienced some episodes of AHA, which were managed at outside institutions; in particular, a severe episode of AHA, probably triggered by urinary infection and antibiotics [5], had complicated her second and last delivery. The hemolytic episodes were self-limiting and resolved without sequelae. No other causes of hemolysis were documented. When the case came to our attention, a diagnosis of ITP was made; hemolytic parameters were normal, although the G6PD enzyme concentration was not measured. Oral prednisone (1 mg/kg) was given with only a transient benefit. The patient was then a candidate for elective splenectomy. However, given her extremely low platelet count, she was started in October 2014 on eltrombopag at 50 mg/day as a bridge to splenectomy. Given that, to the best of our knowledge, the use of this drug has never been reported in the particular setting of G6PD deficiency, the patient was constantly monitored. A prompt platelet increase (178x109/L) was observed 1 week after the start of treatment. After she achieved the target platelet count, the dose of eltrombopag was tapered to the lowest effective dose. The patient’s response was stabile while she remained on a dose of eltrombopag between 25 and 50 mg/day without any adverse events; in particular, no variations of hemolytic parameters were observed. As of today, after 6 months of continuous eltrombopag administration, the patient has constantly maintained the target platelet counts and she is awaiting elective splenectomy. According to our knowledge, we report for the first time the evidence regarding the safe use of this thrombomimetic agonist, which provided an excellent treatment outcome without any adverse effects, in a steroid-refractory adult ITP patient at risk of drug-induced AHA as a G6PD-deficient heterozygous carrier. G6PD deficiency is an X-linked, hereditary genetic defect [2,3,4] for which heterozygous women, who are usually asymptomatic, have a mosaicism of normal and G6PD-deficient erythrocytes. Given the possible decreased amount of G6PD enzyme, although exceptional and only under particularly stressing conditions, such as urinary tract infections and/or the use of certain antibiotics such as nitrofurantoin, AHA may occur [5]. Although our report, being limited to a single patient, is purely anecdotal, considering the high prevalence of G6PD deficiency and the increasing use of thrombomimetic drugs, further collection of such cases would be very useful to determine the complication risks associated in this setting with the use of thrombopoietin agonists.
